# Plasma microRNA expression in adolescents and young adults with endometriosis: the importance of hormone use

**DOI:** 10.3389/frph.2024.1360417

**Published:** 2024-04-11

**Authors:** Paula Brady, Abdelrahman Yousif, Naoko Sasamoto, Allison F. Vitonis, Wojciech Fendler, Konrad Stawiski, Mark D. Hornstein, Kathryn L. Terry, Kevin M. Elias, Stacey A. Missmer, Amy L. Shafrir

**Affiliations:** ^1^ Columbia University Fertility Center, Department of Obstetrics and Gynecology, Columbia University Irving Medical Center, New York, NY, United States; ^2^ Department of Obstetrics and Gynecology, Texas Tech University Health Sciences, El Paso, TX, United States; ^3^ Department of Obstetrics and Gynecology, Brigham and Women's Hospital and Harvard Medical School, Boston, MA, United States; ^4^ Boston Center for Endometriosis, Boston Children’s Hospital and Brigham and Women’s Hospital, Boston, MA, United States; ^5^ Department of Biostatistics and Translational Medicine, Medical University of Łódź, Łódź, Poland; ^6^ Department of Epidemiology, Harvard T.H. Chan School of Public Health, Boston, MA, United States; ^7^ Division of Adolescent and Young Adult Medicine, Department of Pediatrics, Boston Children’s Hospital and Harvard Medical School, Boston, MA, United States; ^8^ Department of Obstetrics, Gynecology, and Reproductive Biology, College of Human Medicine, Michigan State University, Grand Rapids, MI, United States; ^9^ Department of Nutrition & Public Health, School of Nursing and Health Sciences, Merrimack College, North Andover, MA, United States

**Keywords:** endometriosis, microRNA, exogenous hormones, adolescents, diagnostic

## Abstract

**Introduction:**

Prior studies have investigated the diagnostic potential of microRNA (miRNA) expression profiles for endometriosis. However, the vast majority of previous studies have only included adult women. Therefore, we sought to investigate differential expression of miRNAs among adolescents and young adults with endometriosis.

**Methods:**

The Women's Health Study: from Adolescence to Adulthood (A2A) is an ongoing WERF EPHect compliant longitudinal cohort. Our analysis included 64 patients with surgically-confirmed endometriosis (96% rASRM stage I/II) and 118 females never diagnosed with endometriosis frequency matched on age (median = 21 years) and hormone use at blood draw. MicroRNA measurement was separated into discovery (10 cases and 10 controls) and internal replication (54 cases and 108 controls) phases. The levels of 754 plasma miRNAs were assayed in the discovery phase using PCR with rigorous internal control measures, with the relative expression of miRNA among cases vs. controls calculated using the 2^−ΔΔCt^ method. miRNAs that were significant in univariate analyses stratified by hormone use were included in the internal replication phase. The internal replication phase was split 2:1 into a training and testing set and utilized FirePlex miRNA assay to assess 63 miRNAs in neural network analyses. The testing set of the validation phase was utilized to calculate the area under the curve (AUC) of the best fit models from the training set including hormone use as a covariate.

**Results:**

In the discovery phase, 49 miRNAs were differentially expressed between endometriosis cases and controls. The associations of the 49 miRNAs differed by hormone use at the time of blood draw. Neural network analysis in the testing set of the internal replication phase determined a final model comprising 5 miRNAs (miR-542-3p, let-7b-3p, miR-548i, miR-769-5p, miR-30c-1-3p), yielding AUC = 0.77 (95% CI: 0.67–0.87, *p* < 0.001). Sensitivity in the testing dataset improved (83.3% vs. 72.2%) while the specificity decreased (58.3% vs. 72.2%) compared to the training set.

**Conclusion:**

The results suggest that miR-542-3p, let-7b-3p, miR-548i, miR-769-5p, miR-30c-1-3p may be dysregulated among adolescent and young adults with endometriosis. Hormone use was a significant modifier of miRNA dysregulation and should be considered rigorously in miRNA diagnostic studies.

## 1 Introduction

Endometriosis, the implantation of endometrial-like glands and stroma outside of the uterus, affects approximately 10% of reproductive-age women (1, 2). Endometriosis is associated with pelvic pain, dyspareunia, dysmenorrhea, bladder and bowel dysfunction, fatigue, and infertility (3). Currently, the standard method of diagnosis is laparoscopic surgery, which creates barriers to care, delays diagnosis, introduces risk of surgical complications, and requires absence from school or work (4). Radiologic imaging is sensitive and specific, but only when endometrioma, deep lesions, or revised American Society for Reproductive Medicine (rASRM) stage III/IV disease is present (4). The average delay from symptom onset to diagnosis is approximately 7 years and over 50% of adults with endometriosis report that their symptoms began during adolescence (5, 6). Given these barriers and the potentially etiologically important time period of adolescence and young adulthood among whom rASRM stage I/II disease is dominant, there is a great need to identify biomarkers to serve as a non-invasive diagnostic for endometriosis among adolescents and young adults.

MicroRNAs (miRNAs), which are small non-coding RNAs involved in epigenetic gene regulation largely through messenger RNA silencing, are an attractive possibility for such a biomarker (7, 8). These miRNAs have been identified in a wide variety of biologic samples, including tissues, serum, plasma, saliva, and urine. Compared to other RNA species, miRNAs are relatively RNAase-resistant, rendering them more stable against degradation (9). Recently research has expanded into elucidating miRNA profiles of endometriosis (10–12), and investigating how changes in miRNA expression may impact gene expression in ectopic endometrial tissue (13, 14). While recent studies have reported promising results for the use of miRNA signatures in endometriosis diagnosis (15–19), gaps remain in our understanding of the relationship between miRNAs and endometriosis etiology (12). In particular, previous studies of miRNA diagnostic potential have been conducted among adult women with endometriosis (15, 16, 18–22); however, there is a lack of information regarding miRNA levels among adolescents with endometriosis.

Identifying endometriosis at an earlier age may help to prevent later life morbidity caused by the condition. Unfortunately, considerable heterogeneity has been reported across studies in terms of the diagnostic potential of individual miRNAs. A recent review by Leonova et al. (2021), noted that 63 miRNAs were reported to be differentially expressed between women with and without endometriosis across 18 studies; however, only 14 of those miRNAs were reported in more than one study (10). Currently, miRNA-based diagnostic tests are in active development for use in patient care across a variety of disease outcomes, and some are already available for clinical use including a saliva-based miRNA test for endometriosis that showed promising interim results (16, 23, 24). However, before these tests can be offered to a wide population, it is critical that we understand how these miRNA-based diagnostic tests may apply in different populations, including varied age groups and accounting for factors such as hormonal medication use.

Current barriers to diagnosis of endometriosis generate significant delays in patient identification and treatment; this is particularly relevant for adolescent patients where early intervention has been shown to slow disease progression, which may improve fertility and functional outcomes (25, 26). The primary goal of this research was to identify miRNAs that differ between adolescent endometriosis cases and controls in a discovery phase, and then in an internal replication phase, test those miRNAs and build a predictive model for endometriosis, using age- and hormone-matched controls among adolescents and young adult women.

## 2 Methods and materials

### 2.1 Study population

The Women's Health Study: From Adolescence to Adulthood (A2A) is a longitudinal observational cohort study that enrolled 1,549 participants [*n* = 787 surgically confirmed women with endometriosis (cases) and *n* = 762 controls] from November 2012–June 2018, enrolling 85% of those who were eligible to participate in the study. Details of the study have been described previously (27, 28). In brief, participants with endometriosis were enrolled from (1) patients with a surgical diagnosis of endometriosis at Boston Children's Hospital (BCH) or Brigham and Women's Hospital (BWH) or (2) from patients with previous documented surgical diagnosis elsewhere but who were receiving follow-up treatment at BCH or BWH. Controls, women without a known diagnosis of endometriosis, were identified through local clinics, local advertisements, online postings, or word of mouth to ensure sampling from the communities served by these two hospitals and thus the underlying population that gave rise to the case participants (29). These controls are representative of the underlying general population. All participants could have been receiving standard of care for any medical conditions including pelvic pain. Of the 762 controls, 82% were community-based and 18% were clinic-based controls. This study was approved by the BCH Institutional Review Board on behalf of both BCH and BWH. Written informed consent was obtained, with both parental consent and participant assent for individuals aged <18 years at enrollment.

All participants, regardless of endometriosis status, were asked to complete an extensive baseline questionnaire and annual follow-up questionnaires. The initial version of the baseline questionnaire assessed demographics, body mass index (BMI), physical activity, diet, smoking, alcohol consumption, reproductive factors, and other medical conditions as well as details on pain symptoms, treatment regimen, and medication use. In January 2014, an expanded version of the World Endometriosis Research Foundation (WERF) Endometriosis Phenome and Biobanking Harmonization Project (EPHect) clinical questionnaire (30) was adopted for use at baseline, although there was very little change in the questionnaire with most of the questions being the same. Surveys were collected and managed with the use of REDCap electronic data capture tools (31). Surgical details for endometriosis cases were documented using the WERF EPHect surgical form (32).

### 2.2 Analytic sample selection

A small sample set was first selected for the discovery phase of the project and a larger sample set was assembled for the internal replication phase. In both phases, endometriosis cases and controls with plasma samples were frequency matched on age (within two years) and hormone use at the time of sample collection (any/none). The discovery phase was limited to participants aged 15–19 years while the internal replication phase included participants aged 13–25 years. Participants with endometriosis and blood samples collected within 6 months after surgery were excluded due to possible perturbations of miRNAs in the post-operative healing period. In the discovery phase of this study, further exclusion criteria were imposed to eliminate any confounding conditions that may affect miRNA profiles, therefore patients with autoimmune or inflammatory conditions (i.e., Hashimoto's, Graves, Sjogren's, and Crohn's diseases, rheumatoid arthritis, multiple sclerosis, ulcerative colitis, lupus erythematosus, scleroderma and psoriasis), and those less than 2 years from menarche (due to immaturity of the hypothalamic-pituitary-gonadal axis and irregular cycles) were excluded.

Twenty participants (10 endometriosis cases and 10 controls) were included in the discovery phase and 162 (54 endometriosis cases and 108 controls) were included in the internal replication phase.

### 2.3 Blood collection

Blood samples were collected at enrollment in accordance with WERF EPHect protocols with the exception of centrifuge speed (33). Whole blood samples were collected in tubes with EDTA or heparin, with 100% of the Discovery phase and 88% of the Internal replication phase collected in EDTA tubes. Blood samples were centrifuged at 1,790 × g for 10 min at 4°C; the plasma was aspirated, aliquoted into cryovials, then frozen to −80°C. Eighty-five percent of samples were processed within 5 h of collection. A biospecimen questionnaire was asked at the time of blood collection, assessing information such as medication use including hormones and pain medications at blood draw.

### 2.4 Covariates

Demographic and anthropometric characteristics of the participants include age (years), baseline body mass index (BMI), race and Hispanic ethnicity, working/education status, and age at menarche (years). BMI was calculated from self-reported weight and height. For women aged ≥20 years, participants were categorized as underweight (BMI < 18.5 kg/m^2^), normal-weight (BMI 18.5–24.9 kg/m^2^), overweight (BMI 25–29.9 kg/m^2)^, or obese (BMI ≥ 30 kg/m^2^) according to World Health Organization (WHO) criteria (34). For participants <20 years, the age- and gender-specific BMI Z-score was calculated and categorized as underweight (Z-score, ≤−2), normal weight (Z-score, >−2–<1), overweight (Z-score, 1–2), or obese (Z-score, >2). Other exposures included type of hormone use at the time of blood draw and pain medications used in the 48 h prior to blood draw (any, none). Pain symptoms of interest included acyclic and cyclic (i.e., dysmenorrhea) pelvic pain. The severity of each type of pelvic pain at its worst was rated on a 0–10 numeric rating scale (NRS), with 0 corresponding to no pain and 10 to the worst imaginable pain.

### 2.5 Discovery phase assay

Total RNA was isolated from the plasma samples of 10 adolescent participants with endometriosis and 10 matched controls using the mirVana Paris kit (Thermo Fisher Scientific, Waltham, MA). Each RNA sample was assessed for quality and miRNA concentration using the Agilent 2,100 Bioanalyzer (Agilent Technologies, Santa Clara, CA).

Real-time qPCR was performed in two steps. Complementary DNA (cDNA) were reverse transcribed using miRNA primers and reagents from the TaqMan MicroRNA Reverse Transcription Kit (Thermo Fisher Scientific, Waltham, MA). PCR products were then amplified using the TaqMan MicroRNA Assay and TaqMan Universal PCR Master Mix (Thermo Fisher Scientific, Waltham, MA). miRNA expression was measured for each sample in technical duplicate using TaqMan Low Density Array Human MicroRNA A + B Cards v3 (Life Technologies, Inc., Carlsbad, CA) to measure expression of 754 human miRNAs based on miRBase v20. All PCR reactions were performed on a 7,500 Real-Time PCR system (Applied Biosystems, Foster City, CA). miRNAs of interest were selected for further investigation in the internal replication phase based on univariate analysis stratified by hormone use.

### 2.6 Internal replication phase assay

Internal replication of the dysregulated miRNAs identified in the discovery phase was performed using the FirePlex™ MicroRNA Assay (Abcam, Cambridge, MA), using a new set of 162 samples (54 adolescents and young adults with endometriosis and 108 age and hormone frequency matched controls). In addition to the 49 miRNAs identified in the discovery phase (see Statistical Analysis section for details on miRNA selection), additional probes were included to cover miRNAs associated with endometriosis in previous published reports (*n* = 8) plus Fireplex positive control (let-7g-3p, let-7d-3p, and miR-29b-3p) and non-human species negative control (ath-miR-167d, oan-miR-7417-5p, and cel-miR-70-3p) miRNAs recommended by Abcam to create a final set of 63 probes.

Each sample well of the 96-well plate contained probes to every miRNA to be measured. In addition to case and control samples, pooled human plasma was included as blinded quality control samples (representing 5% of the total sample number). Plates also contained blank wells with water controls to measure background fluorescence. Samples were processed using the FirePlex™ MicroRNA Assay as per protocol. By the nature of this assay, miRNA extraction was not required and the initial hybridization step removed heparin from the samples collected in heparin tubes, thereby eliminating any potential effects of heparin on the assay results (35). RNA samples were hybridized to miRNA-specific probes in hydrogel beads, followed by ligation to a universal biotinylated adaptor labelled with a fluorescent reporter. The level of fluorescence corresponds to the amount of miRNA in the plasma sample, detected using a Guava® 6HT flow cytometer (MilliporeSigma, Burlington, MA). The flow cytometer standard (FCS) files were analyzed with the FirePlex™ Analysis Workbench software.

### 2.7 Statistical analyses

#### 2.7.1 Discovery phase

In the discovery phase, PCR analyses were performed within GeneEx v.6 (MultiD Analyses AB, Goteborg, Sweden) in compliance with MIQE guidelines (36). During pre-processing, missing values were imputed and outliers were identified and excluded. miRNAs with a cycle threshold (Ct) value greater than 34 were excluded. Global normalization was performed across all samples, then the Normfinder algorithm was used to select the most stable miRNAs across all samples to estimate relative fold changes (37, 38). This identified miR-146b, miR-152, miR-185, miR-301, and miR-590-5p as the best reference miRNA sequences for this dataset. The relative expression of miRNA among cases vs. controls was calculated using the 2^−ΔΔCt^ method (39). Student's *t*-tests were utilized to select miRNAs for inclusion in the internal replication phase. Analyses were conducted separately by hormone use status (any/none). miRNAs with a *p*-value of <0.005 in the analyses among hormone users or the analyses among non-hormone users were included for analysis in the internal replication phase. Additionally, we included miRNAs with a *p*-value of ≤0.05 in both the analyses among the Hormone users and the analyses among the non-hormone users.

#### 2.7.2 Internal replication phase

To construct predictive models to estimate risk of endometriosis utilizing the 63 miRNAs included in the internal replication phase, the 162 samples were randomized 2:1 into at training set for model development (36 cases and 72 controls) and a testing set for model validation (18 cases and 36 controls). In the training set, the mean fluorescence intensity (MFI) data were log_10_-transformed to obtain normal distributions. Any negative values were shifted to 0.001 prior to log-transformation. We then began with univariate analyses, performing *t*-tests to assess fold-changes in miRNA expression between cases and controls. Correction for multiple testing was performed using a Holm-Sidak post-hoc test (40). Principal components analysis (PCA) was used to estimate the degree of separation between endometriosis cases and controls. As the dataset included more miRNAs than the number of endometriosis cases, we reduced the number of miRNAs to input into the model by preselecting the miRNAs for classification model development using correlation-based feature selection (CFS) analysis (41). CFS is a dimensionality reduction technique whose central hypothesis is that good feature sets contain features that are highly correlated with the class, yet uncorrelated with each other. Subsets of features that are highly correlated with the class while having low inter-correlation with one another are preferred in this process. Models are then built using these classes as variables instead of including all covariates. Within the CFS analyses, we additionally included hormone use as a covariate. CFS was performed with 10-fold cross validation. All miRNAs with an unadjusted *p* ≤ 0.15 in univariate analysis were included.

Models were then built on the training dataset using neural network analysis following the BFGS (Broyden-Fletcher-Goldfarb-Shanno) algorithm as previously described (42). Unlike traditional regression techniques, this machine learning approach can learn and model complex, non-linear relationships. There are no restrictions on the input variables or assumptions on their distributions. Briefly, the network structures consist of a multilayer perceptron with a number of neurons in the hidden layer iteratively optimized from (*n* variables)/3 to (*n* variables)*1.5 to avoid overfitting. Admissible linking functions between the neuron layers can be linear, logistic, hyperbolic tangential, or exponential. In total, we constructed 15,000 networks. We identified the 50 best networks in terms of performance among participants in the training set. We then validated these models among the participants in the testing set using area under the receiver-operator characteristic curves (AUC). In each model, the output represents the probability of endometriosis in a given sample for a certain pattern of expression of miRNAs plus hormone use data. The final model represents the best model performance on the testing set. The AUC was compared against a model curve with an AUC of 0.5 to evaluate the null hypothesis of no predictive value vs. chance alone using the method of Hanley and McNeil (43).

In the internal replication phase, we performed a sensitivity analysis excluding endometriosis cases without pathology results or pathology without endometriosis in the biopsy. This sensitivity analysis reduced the number of cases in the training set to 22. The same number of controls remained.

## 3 Results

The demographic characteristics of endometriosis case and control participants in the discovery and internal replication phases of the study are shown in Table 1. The median age of cases and controls in the discovery phase was 17 and 18.5 years, and in the internal replication phase was 20 and 22 years, respectively. All of the discovery phase endometriosis cases had rASRM stage I/II disease, while 96% of the internal replication phase had rASRM stage I/II disease and the remaining 4% had stage IV disease. Fifty percent of endometriosis cases and controls were taking hormones at the time of blood collection in the discovery set and 78% were taking hormones in the internal replication set. Hormones used were either progesterone only or estrogen and progesterone.

**Table 1:** Participant characteristics by study phase and endometriosis status.

	Discovery phase		Internal replication phase	
	Cases (*n* = 10)	Controls (*n* = 10)	Cases (*n* = 54)	Controls (*n* = 108)
Age (years)				
Median (range)	17 (15–19)	19 (16–19)	20 (13–25)	22 (13–25)
Age at menarche (years)^a^				
Median (range)	12 (11–14)	12 (9–14)	12 (8–15)	12 (9–15)
Race, *n* (%)				
Asian	0 (0%)	1 (10%)	0 (0%)	11 (10%)
Black	0 (0%)	1 (10%)	1 (2%)	5 (5%)
White	10 (100%)	5 (50%)	48 (89%)	84 (78%)
Other	0 (0%)	3 (30%)	5 (9%)	8 (7%)
Ethnicity, *n* (%)				
Hispanic	1 (10%)	1 (10%)	7 (13%)	9 (8%)
Non-Hispanic	9 (90%)	9 (90%)	47 (87%)	99 (92%)
Working/educational status, *n* (%)				
Middle/high school	5 (50%)	2 (20%)	7 (13%)	4 (4%)
College/Grad school	3 (30%)	3 (30%)	22 (41%)	35 (32%)
Working	1 (10%)	3 (30%)	15 (28%)	65 (60%)
Other	1 (10%)	2 (20%)	10 (19%)	4 (4%)
Body mass index, *n* (%)^b^				
Underweight	0 (0%)	0 (0%)	2 (4%)	3 (3%)
Normal weight	8 (80%)	9 (90%)	25 (46%)	80 (74%)
Overweight	2 (20%)	1 (10%)	16 (30%)	21 (19%)
Obese	0 (0%)	0 (0%)	11 (20%)	4 (4%)
Surgery for endometriosis at time of blood draw				
No, past surgery^c^	0 (0%)	–	9 (17%)	–
Yes, diagnostic surgery	9 (90%)	–	25 (46%)	–
Yes, subsequent surgery	1 (10%)	–	20 (37%)	–
Hormone use at time of blood collection, *n* (%)				
No	5 (50%)	5 (50%)	12 (22%)	24 (22%)
Yes	5 (50%)	5 (50%)	42 (78%)	84 (78%)
Pain medications used in past 24–48 h before blood collection, *n* (%)^d^				
No	9 (100%)	9 (90%)	40 (74%)	84 (78%)
Yes	0 (0%)	1 (10%)	14 (26%)	24 (22%)
Severity of dysmenorrhea^e^				
None	0 (0%)	3 (30%)	0 (0%)	24 (22%)
Mild	1 (10%)	3 (30%)	2 (4%)	54 (50%)
Moderate	4 (40%)	2 (20%)	7 (13%)	16 (15%)
Severe	3 (30%)	1 (10%)	13 (24%)	6 (6%)
Not cycling	2 (20%)	1 (10%)	32 (59%)	8 (7%)
Severity of dysmenorrhea in past 12 months^f,g,h^				
Median (range)	8 (6–9)	4 (1–8)	9 (4–10)	4 (1–10)
Acyclic pelvic pain in past 3 months				
No	4 (40%)	9 (90%)	18 (33%)	93 (86%)
Yes	6 (60%)	1 (10%)	36 (67%)	15 (14%)
Acyclic pelvic pain severity in past 3 months^h,i^				
Median (range)	8 (4–8)	4 (4–4)	8 (2–10)	4 (1–8)

^a^ One endometriosis case in internal replication phase missing age at menarche.

^b^ For women aged ≥20 years: underweight (BMI < 18.5 kg/m^2^), normal weight (BMI 18.5–24.9 kg/m^2^), overweight (BMI 25–29.9 kg/m^2^), or obese (BMI ≥ 30 kg/m^2^) according to World Health Organization criteria; For those <20 years, the age- and gender-specific BMI Z-score was calculated, and participants were categorized as underweight (Z-score ≤ −2), normal weight (Z-score > −2–<1), overweight (Z-score 1–2), or obese (Z-score > 2).

^c^ These 9 participants in the internal replication phase had a surgery more than 12 months prior to their enrollment into the A2A cohort. Their blood sample was collected more than 6 months after their last endometriosis-related surgery.

^d^ One case in the discovery phase missing pain medication use at time of blood draw.

^e^ Participants categorized as “not cycling” reported not having menstrual periods in the previous 3 months.

^f^ Among participants who reported mild, moderate or severe dysmenorrhea.

^g^ Three controls in internal replication phase missing severity of dysmenorrhea in past 12 months.

^h^ Based on 0–10 numeric rating scale with 0 = no pain and 10 = worst pain imaginable.

^i^ Among participants who reported acyclic pelvic pain in the past 3 months.

### 3.1 Discovery phase

The discovery phase identified 5 miRNAs differentially expressed in cases relative to controls that were nominally significant (*t*-test *p*-values ≤ 0.05), irrespective of hormone use, in addition to 44 miRNA dysregulated in either hormone users (*n* = 21) or non-users (*n* = 23) (*p* < 0.005) (Table 2, Supplementary Tables S1, S2). All dysregulated miRNAs with nominal significance had a relative fold change of at least 2 (absolute value mean 20, range 2–336).

**Table 2:** Dysregulated miRNA by hormone status in the discovery set (*n* = 10 cases and 10 controls).

Dysregulated miRNA in both hormone users and non-hormone users				
miRNA	Hormone users		Non-hormone users	
	Fold change	*p*-value	Fold change	*p*-value
miR-548l	−9.53	0.007	2.57	0.04
miR-1296-5p	−7.18	0.05	−18.99	0.02
let-7i-3p	3.88	0.05	3.45	0.03
miR-651-5p	−3.56	0.002	−6.03	0.04
miR-33a-5p	2.51	0.02	12.06	0.004
Dysregulated miRNA only in hormone users, miRNA (fold change)				
miR-626 (−75.9); miR-1298-5p (46.8); miR-125b-1-3p (−25.8); miR-589-5p (−22.1); miR-542-3p (−20.6); miR-122-3p (19.4); miR-337-3p (−19.3); miR-219a-5p (−18.0); miR-376b-3p (−13.9); miR-422a (12.7); let-7a-3p (−12.0); miR-567 (−10.1); miR-193a-3p (−8.6); miR-124-3p (−7.9); miR-30c-1-3p (−6.4); miR-891a-5p (−4.5); miR-23b-5p (−3.9); miR-935 (3.8); miR-219-1-3p (2.8); miR-154-5p (−2.4); miR-455-5p (2.1)				
Dysregulated miRNA only in non-hormone users, miRNA (fold change)				
miR-588 (−335.8); let-7b-3p (−122.0); let-7c-5p (−55.3); miR-541-3p (−20.1); miR-641 (−16.1); miR-33b-5p (14.9); miR-296-3p (14.5); miR-500a-3p (−11.7); miR-192-3p (8.9); miR-200a-5p (−8.8); miR-519d-3p (7.7); miR-106a-3p (6.1); miR-548E-3p (−5.8); miR-127-5p (−5.5); miR-548a-5p (−5.0); miR-548 K (4.7); miR-29b-1-5p (4.3); miR-147a (4.1); miR-548i (−4.0); miR-544a (3.5); miR-504-5p (−2.3); miR-769-5p (−2.0); miR-153-3p (−2.0)				

### 3.2 Internal replication phase

**Figure 1:**
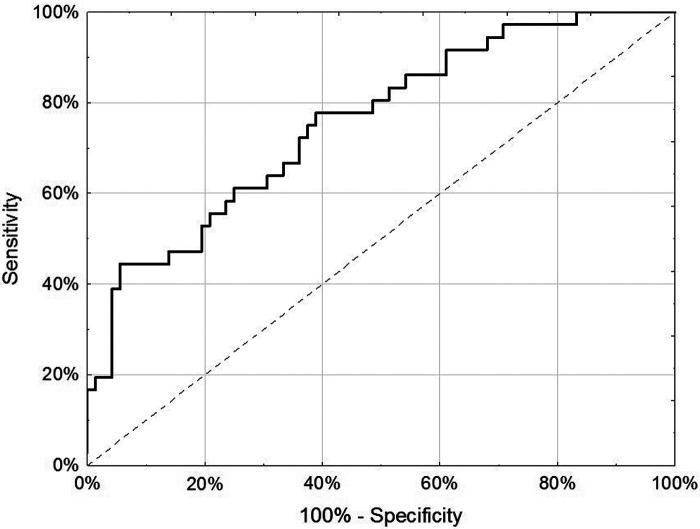
Receiver-operator characteristic curve of 5 miRNA (miR-542-3p, let-7b-3p, miR-548i, miR-769-5p and miR-30c-1-3p) and hormone use (any/none) in discrimination of endometriosis cases from controls in the testing dataset of the internal replication phase.

For the internal replication phase, in addition to the 49 miRNAs identified in the discovery phase, 8 additional miRNAs were added based on literature review and 6 internal controls to yield a final list of 63 miRNAs (Supplementary Table S3). While the miRNA relative fold changes were all above 2 in the discovery phase, in the internal replication phase training dataset, only one miRNA had a relative fold change >2. When we restricted the endometriosis case definition to only those with pathologically confirmed endometriosis, three miRNA had relative fold changes >2. When applied to the internal replication training dataset, univariate analyses were insufficient for discrimination between cases and controls. No individual miRNA had *p*-value < 0.05 when correcting for multiple testing definition (Supplementary Table S4). Similarly, neither PCA nor unsupervised clustering analyses could distinguish the cases from controls (Supplementary Figures S1, S2). Given the finding in the discovery set that most miRNA differences between endometriosis cases and controls were specific to either hormone users or non-users, we suspected that hormone use was a potentially mediating or modifying variable and forced it to be included in the model development. To identify the best set of miRNAs including hormone use that discriminates endometriosis cases from controls, we elected to follow an approach we have previously employed in miRNA analyses and used neural network analysis (42). Performance of the neural network in both the training and testing datasets is shown in Table 3. This modeling technique yielded a final model comprising 5 miRNAs (miR-542-3p, let-7b-3p, miR-548i, miR-769-5p, miR-30c-1-3p) and hormone status (Figure 1). When applied to the testing dataset, the AUC for this model was 0.77 (95% CI: 0.67–0.87, *p* < 0.001). Compared to the training dataset, the sensitivity in the testing dataset improved (83.3% vs. 72.2%) while the specificity decreased (58.3% vs. 72.2%; Table 3).

**Table 3:** Performance of the neural network model in adolescents and young women, including plasma miRNAs miR-542-3p, let-7b-3p, miR-548i, miR-769-5p, and miR-30c-1-3p and including hormone use as a covariate.

Dataset	True positives	False positives	False negatives	True negatives	Sensitivity	Specificity
					(95% CI)	(95% CI)
Training Set	26	20	10	52	72.2 (54–86%)	72.2 (60–82%)
Testing Set	15	15	3	21	83.3 (58–96%)	58.3 (41–74%)

## 4 Discussion

Using two different platforms in separate discovery and internal replication phases, this study identified a model including 5 miRNAs (miR-542-3p, let-7b-3p, miR-548i, miR-769-5p, miR-30c-1-3p) plus hormone use status that could distinguish adolescents and young adults with endometriosis from age-matched controls with high sensitivity but low specificity. Hormone use at the time of blood draw appeared to be a significant modifier of miRNA expression. Uniquely this study included adolescents and young adults, which is an understudied population for miRNA discovery even though more than 50% of endometriosis patients report that their symptoms started in adolescence (5).

The miRNA identified in this study have plausible involvement in pathogenesis and pathophysiology of endometriosis. Four of the miRNAs we found to be downregulated in adolescents and young women with endometriosis (i.e., Let-7b-3p, miR-542-3p, miR-769-5p, miR-30c-1-3p); downregulation of these miRNA is associated with enhanced cell proliferation, migration, invasion and reduced apoptosis. Let-7b-3p has been shown to target Polo-like kinase 1 (PLK1), thereby inhibiting cell proliferation and enhancing apoptosis (44). Aberrant miR-542-3p expression has been identified in a variety of malignancies, and miR-542-3p may function as a tumor suppressor, including in ovarian cancer (45, 46). Downregulation of miR-542-3p has been reported in another study of miRNA in the sera of adults with endometriosis (47). MiR-769-5p has been found to suppress cell proliferation, migration and invasion, by silencing Transforming Growth Factor Beta Receptor 1 (TGFBR1) (48). TGFBR1 has been found to be upregulated in the walls of endometriomas, and promotes fibrosis (49). MiR-30c-1-3p has been shown to suppress metastasis of gastrointestinal stromal tumors, and is associated wth prevention of prostate cancer progression by modulating cell proliferation via androgen receptor downstream targets (50, 51). More broadly, MiR-30c-1-3p has been shown to silence the pregnane X receptor, which has been implicated in the development of a variety of malignancies and metabolic disorders (52). While miR-548i was also downregulated, function of miR-548i is limited, and may have novel biologic activities which warrant future research.

Previous studies investigating miRNA levels in blood samples from endometriosis patients have reported varying results (10–12). Of the more recent studies, multiple blood-based miRNAs have been included in diagnostic models of endometriosis; however, none of the potential diagnostic miRNAs identified overlapped between studies (17–20, 22, 53–56). Vanhie et al. (2019) (54), Nisenblat et al. (2019) (22), and Papari et al. (2020) (18) have assessed miRNA diagnostic models for endometriosis among independent validation cohorts. Utilizing blood plasma samples, Vanhie et al. (2019) (54) reported that 42 miRNAs were differentially expressed between endometriosis patients and surgically-disconfirmed controls in their discovery phase; however, none of the associations were significant after correcting for multiple testing. Within the validation cohort, only the diagnostic model including three miRNAs (miR-125b-5p, miR-28-5p, miR-29a-3p) comparing endometriosis patients with rASRM stage I/II disease to controls reached an AUC above 0.5 (AUC = 60%) (54). Similarly, Nisenblat et al. (2019) (22) reported that three miRNAs—miR-139-3p, miR-155, miR-574-3p—were consistently dysregulated in two independent cohorts of participants with endometriosis compared to those without; however, none of these miRNAs or the combination of all three had sufficient sensitivity or specificity to be utilized as a diagnostic test. In contrast, Papari et al. (2020) (18) assessed the diagnostic potential of 24 miRNAs including 20 identified from an earlier preliminary screen. Among 25 endometriosis patients and 28 controls, a miRNA signature with 8 miRNAs (miR-199a-3p, miR-143-3p, miR-340-5p, let-7b-5p, miR-21-5p, miR-17-5p, miR-20a-5p, miR-103a-3p) resulted in an AUC of 0.95 with a sensitivity of 0.92 and specificity of 0.86, which is similar to the discrimination ability of laparoscopic surgery (18). Differences between previous studies and our results could be due to (1) differences in control populations selected as alternations in miRNA levels may differ between other benign gynecologic conditions that control participants may have had, (2) differences in participant age as dysregulation of miRNAs may differ at different points in endometriosis pathophysiology, and (3) differences due to hormone use among study participants as miRNA expression appears to differ by hormone use status.

Similar to some of the previous studies, we observed downregulation of miRNAs let-7b-3p (57, 58) and miR-542-3p (47). Disparate from these previous studies, we observed downregulation of miRNAs miR-769-5p and miR-30c-1-3p, which have not been extensively studied in endometriosis patients. However, our study population included participants who were younger than previous studies, which may explain differences observed compared to previous studies. Additionally, among our endometriosis case participants, 96% had documented rASRM stage I/II, with only two patients staged III/IV. Further, all endometriosis participants were pain presenting at diagnosis and none of them had experienced infertility. As the vast majority of diagnostic miRNA studies have been conducted among adult endometriosis patients, there is a need to expand miRNA research to include adolescents in order to identify endometriosis at earlier stages.

In addition, our study accounted for hormone exposure at blood collection, which was determined to drive significant heterogeneity in miRNA associations with endometriosis. In contrast, Nisenblat et al. (2019) assessed changes in miRNA levels by menstrual cycle phase (early follicular, late follicular, luteal) among 8 women with endometriosis and 8 healthy controls and did not note any substantial differences in miRNA levels by menstrual cycle phase (22). This difference could be due to different physiological influences of a natural menstrual cycle and exogenous hormone use on miRNA expression or underpowered analyses given the small sample size included for assessing changes across the menstrual cycle. Further, the majority of previous studies have restricted to participants who did not use hormonal treatments in the past 3 months (18, 19, 55, 56); however, we observed an important modifying or mediating effect of hormone use within our adolescent and young adult population for identifying miRNAs that are associated with endometriosis. As patients with endometriosis symptoms frequently use hormonal treatments before surgical confirmation of their diagnosis, it is important that future studies take into account hormone use for an improved understanding how these diagnostic tests may be utilized in the future.

Recent research has also included the identification of miRNAs for endometriosis that can be measured in saliva due to the ease of accessibility and collection of saliva compared to other bodily fluids (16, 59). Bendifallah et al. (2022) (59) examined the salivary levels of miRNAs in participants older than 18 years presenting with chronic pelvic pain. Participants were diagnosed with endometriosis via laparoscopic surgery or MRI imaging if laparoscopy was not performed. Among 153 endometriosis participants and 47 controls, a model of 109 miRNAs had sensitivity, specificity, and AUC of 96.7%, 100%, and 98.3%, respectively (59). Recently interim results of an external validation of the 109 miRNA signature yielded similar results for sensitivity, specificity, and AUC; however, these interim results were only based on 159 endometriosis cases and 41 controls and does not include adolescents with endometriosis (16). Saliva presents another promising source for discovering a reliable, easily collected diagnostic biomarker for endometriosis; however, caution should be practiced before these results are generalized to other populations, i.e., adolescents and before there is an understanding of how hormonal treatments and other factors may impact on levels of salivary miRNAs.

The present study has multiple strengths. While most prior studies have focused on adult women, this study was novel in its focus on a large sample size of adolescents and young women and is one of the largest studies to date on miRNA in relation to endometriosis. This population of endometriosis patients may have a biologically different disease than adults, or at the very least, represent a unique opportunity for early intervention for the protection of future fertility and functional outcomes (25, 26). While prior studies are limited by variable biologic sample collection, processing, and storage techniques, the clinical data and biologic samples in this study were collected in accordance with the WERF EPHect guidelines (30, 32, 33). This approach not only ensured the scientific rigor of our findings, but also allows for better comparison among future studies. Additionally, the biologic sample selected for this study was plasma, which has some advantages over serum for miRNA analysis in that it is less subject to hemolysis artifact, and which can be obtained non-invasively, unlike eutopic endometrium or surgical specimens (60). Finally, endometriosis patients commonly receive hormonally-active medications, which have been shown to affect miRNA expression (61). In this study, participants’ use of hormonal medications was recorded in detail; this depth and breadth of available data is a major strength of this study, and indeed, was important for the interpretation of miRNA values. Moreover, we were able to account for both hormonal status and the inter-related nature of miRNA using modern machine learning analysis techniques. This approach allows us to examine the impact of more subtle miRNA changes in relation to larger systematic patterns.

The limitations of this study include the performance of the technologies used to assess miRNA expression. All platforms vary in sensitivity, specificity, reproducibility, and accuracy in the detection of miRNA; the combination of Real-Time qPCR and FirePlex, however, can potentially compensate for these variations and optimize the detection of miRNA expression. Additionally, after multiple testing correction, none of the miRNAs from the discovery phase were significant. Further, blood collection was conducted with both EDTA and heparin tubes in the Internal replication phase. Although anticoagulant type can affect miRNA expression (62), the initial hybridization step in the Fireplex functions as an isolation step thereby eliminating any potential effects of heparin on the assay (35). Regarding the control group in this study, while endometriosis is not definitively excluded in these adolescents and young women, it's estimated that approximately 2% of asymptomatic women have endometriosis, which still allows for meaningful utility as a control group (63). The current study concerns adolescents and young women; generalizability to women in their later 20s and beyond is unclear.

Delayed diagnosis of endometriosis negatively impacts patient well-being and inflates health care costs, while early identification of endometriosis and intervention in adolescent patients can slow disease progression, which may improve functional outcomes (25). A blood-based diagnostic test for endometriosis could theoretically allow patients to avoid surgery, facilitating earlier diagnosis and faster direct referral to endometriosis specialists. While the sensitivity and specificity of our miRNA diagnostic signature are modest, the identification of specific miRNAs associated with adolescent and young adult disease may prove useful for pathophysiology discovery. In future studies it will be critical to thoughtfully consider the incorporation of hormonal treatments and other factors that may influence miRNA levels and incorporate these factors into diagnostic model building. Eventually, as targeted therapeutics advance, miRNA profiles of endometriosis may allow for personalized precision medical treatment and may also prove helpful in monitoring for endometriosis activity and recurrence, limiting the need for reoperation.

## Data Availability

The datasets presented in this article are not readily available because data are not publicly available due to information that could compromise research participants’ privacy and consent. However, experienced scientists who would like to inquire regarding use of data from this study to address specific hypotheses or replicate the analyses in this study may submit an application and research proposal. Data requests must be reviewed and approved by the Brigham and Women's Hospital Institutional Review Broad (https://www.brighamandwomens.org/research/research-administration). Data sharing will require a fully executed Data Usage Agreement. Requests to access the datasets should be directed to the A2A cohort leadership committee (womenshealthstudy@bwh.harvard.edu).

## Supplementary material

Supplemental Figure 1 Principal component analysis of endometriosis cases versus controls in the internal replication phase using the 63 miRNAs.

Supplemental Figure 2 Sample classification for miRNA in the internal replication phase. Heatmap shows sample classification for all miRNAs with a nominal *p* < 0.10. Data represent hierarchical clustering by Euclidean distance normalized by row and optimized for layout.
