# Supplementary material for: Plasma microRNA expression in adolescents and young adults with endometriosis: the importance of hormone use
Source: Front Reprod Health. 2024 Apr 11;6:1360417. doi: 10.3389/frph.2024.1360417 (PMC11043576; doi:10.3389/frph.2024.1360417)
Supplement: Supplementary file 1 [file Table1.docx]

**Supplemental Table 1**. miRNA targets comparing endometriosis cases to controls in the discovery phase among hormone users with t-test p-values ≤0.05.^1^

| **miRNA name-primer code** | **Fold change** | **Difference (A-B log scale)** | **P-Value** |
| --- | --- | --- | --- |
| hsa-let-7a-3p-002307 | -12.02288 | -3.58771 | 0.00000002 |
| hsa-miR-122-3p-002130 | 19.41159 | 4.27885 | 0.000002 |
| hsa-miR-626-001559 | -75.893 | -6.2459 | 0.000002 |
| hsa-miR-125b-1-3b-0023 | -25.81805 | -4.69031 | 0.000006 |
| hsa-miR-567-001534 | -10.14531 | -3.34274 | 0.00002 |
| hsa-miR-193a-3p-0022 | -8.57299 | -3.0998 | 0.00006 |
| hsa-miR-154-5p-000477 | -2.35472 | -1.23555 | 0.00006 |
| hsa-miR-30c-1-3p-00210 | -6.4226 | -2.68316 | 0.00008 |
| hsa-miR-455-5p-001280 | 2.11664 | 1.08177 | 0.0001 |
| hsa-miR-935-002178 | 3.81841 | 1.93297 | 0.0001 |
| hsa-miR-422a-002297 | 12.67481 | 3.66389 | 0.0002 |
| hsa-miR-337-3p-00215 | -19.30333 | -4.27078 | 0.0002 |
| hsa-miR-219-1-3p-002 | 2.81359 | 1.49241 | 0.0003 |
| hsa-miR-23b-5p-002126 | -3.92978 | -1.97445 | 0.0005 |
| mmu-miR-124-3p-001182 | -7.86442 | -2.97534 | 0.0006 |
| hsa-miR-589-5p-002409 | -22.12046 | -4.46731 | 0.001 |
| hsa-miR-376b-3p-001102 | -13.87699 | -3.79462 | 0.001 |
| hsa-miR-651-5p-001604 | -3.5603 | -1.832 | 0.002 |
| hsa-miR-1298-5p-002861 | 46.78644 | 5.54802 | 0.002 |
| hsa-miR-891a-5p-002191 | -4.48505 | -2.16512 | 0.003 |
| hsa-miR-219a-5p-000522 | -18.02994 | -4.17232 | 0.003 |
| hsa-miR-542-3p-00128 | -20.61833 | -4.36586 | 0.003 |
| hsa-miR-409-5p-00233 | -4.00748 | -2.00269 | 0.003 |
| hsa-miR-29a#-002447 | 11.39363 | 3.51016 | 0.006 |
| hsa-miR-548L-002904 | -9.53594 | -3.25337 | 0.007 |
| hsa-miR-570-002347 | -10.3437 | -3.37068 | 0.008 |
| hsa-miR-138-2#-00214 | 9.95707 | 3.31572 | 0.008 |
| hsa-miR-512-3p-00182 | 9.89501 | 3.3067 | 0.01 |
| hsa-miR-218-000521 | 693.74486 | 9.43826 | 0.01 |
| hsa-miR-548J-002783 | -2.28936 | -1.19494 | 0.02 |
| hsa-miR-33a-5p-002135 | 2.51243 | 1.32908 | 0.02 |
| hsa-miR-342-3p-00226 | -2.90577 | -1.53892 | 0.02 |
| hsa-miR-548a-001538 | -2.93479 | -1.55326 | 0.02 |
| hsa-miR-429-001024 | 3.23491 | 1.69373 | 0.04 |
| hsa-miR-548d-001605 | -9.08207 | -3.18302 | 0.04 |
| RNU44-001094 | 5.93841 | 2.57008 | 0.04 |
| hsa-miR-551b#-002346 | -21.06126 | -4.39652 | 0.05 |
| hsa-miR-1296-5p-002908 | -7.18323 | -2.84463 | 0.05 |
| hsa-let-7i-3p-002172 | 3.88349 | 1.95735 | 0.05 |

^1^miRNA with a p-value<0.005 were carried into the internal replication phase. miRNA with a p-value <0.05 that also had a p-value of <0.05 in Supplemental Table 2 were also carried into the internal replication phase.
