# Supplementary material for: Plasma microRNA expression in adolescents and young adults with endometriosis: the importance of hormone use
Source: Front Reprod Health. 2024 Apr 11;6:1360417. doi: 10.3389/frph.2024.1360417 (PMC11043576; doi:10.3389/frph.2024.1360417)
Supplement: Supplementary file 2 [file Table2.docx]

**Supplemental Table 2**. miRNA targets comparing endometriosis cases to controls in the discovery phase among those not using hormones with t-test p-values ≤0.05^1^

| **miRNA Name-primer code** | **Fold change** | **Difference (A-B log scale)** | **P-Value** |
| --- | --- | --- | --- |
| hsa-miR-519d-3p-002403 | 7.69655 | 2.94421 | 0.00000001 |
| hsa-miR-588-001542 | -335.78451 | -8.39139 | 0.00000001 |
| hsa-miR-1201-002781 | -125.16959 | -6.96774 | 0.00000001 |
| hsa-miR-106a-3p-002170 | 6.10594 | 2.61021 | 0.00000002 |
| hsa-miR-127-5p-00222 | -5.53567 | -2.46876 | 0.00000003 |
| hsa-miR-192-3p-002272 | 8.93605 | 3.15964 | 0.00000004 |
| hsa-miR-641-001585 | -16.0556 | -4.005 | 0.00000005 |
| hsa-miR-548a-5p-0024 | -4.95833 | -2.30985 | 0.00000008 |
| hsa-miR-296-3p-00210 | 14.54825 | 3.86277 | 0.0000002 |
| hsa-miR-544a-002265 | 3.48552 | 1.80138 | 0.0000002 |
| hsa-let-7c-5p-002405 | -55.28565 | -5.78883 | 0.0000004 |
| hsa-miR-29b-1-5p-00216 | 4.32261 | 2.1119 | 0.000005 |
| hsa-miR-147a-000469 | 4.05656 | 2.02026 | 0.000008 |
| hsa-miR-548E-3p-002881 | -5.81401 | -2.53953 | 0.00006 |
| hsa-miR-541-3p-002201 | -20.10902 | -4.32977 | 0.00008 |
| hsa-miR-500a-3p-001046 | -11.68532 | -3.54662 | 0.00009 |
| hsa-miR-548K-002819 | 4.72183 | 2.23935 | 0.00009 |
| hsa-miR-33b-5p-002085 | 14.86961 | 3.89429 | 0.0004 |
| hsa-let-7b-3p-002404 | -122.04495 | -6.93127 | 0.0004 |
| hsa-miR-504-5p-002084 | -2.28457 | -1.19192 | 0.0006 |
| hsa-miR-548i-002909 | -4.03174 | -2.0114 | 0.0001 |
| hsa-miR-200a-5p-001011 | -8.78973 | -3.13582 | 0.001 |
| hsa-miR-769-5p-00199 | -2.01146 | -1.00824 | 0.001 |
| mmu-miR-153-3p-001191 | -2.00251 | -1.00181 | 0.001 |
| hsa-miR-566-001533 | -54.98649 | -5.78101 | 0.003 |
| hsa-miR-582-3p-00239 | 3.90584 | 1.96563 | 0.004 |
| hsa-miR-33a-5p-002135 | 12.05755 | 3.59187 | 0.004 |
| hsa-miR-204-000508 | 4.74813 | 2.24736 | 0.005 |
| hsa-miR-1247-002893 | 18.0466 | 4.17366 | 0.009 |
| hsa-miR-362-001273 | -2.10124 | -1.07124 | 0.01 |
| hsa-miR-1254-002818 | -2.92988 | -1.55084 | 0.01 |
| hsa-miR-374b#-002391 | 2.57755 | 1.366 | 0.02 |
| hsa-miR-296-000527 | -2.47983 | -1.31024 | 0.02 |
| mmu-miR-491-001630 | -2.14123 | -1.09844 | 0.02 |
| hsa-miR-95-000433 | 11.84462 | 3.56616 | 0.02 |
| hsa-miR-1296-5p-002908 | -18.99055 | -4.24721 | 0.02 |
| hsa-let-7i-3p-002172 | 3.44766 | 1.78562 | 0.03 |
| hsa-miR-886-3p-00219 | -2.82028 | -1.49584 | 0.03 |
| hsa-miR-1233-002768 | -8.18419 | -3.03284 | 0.03 |
| hsa-miR-645-001597 | -4.85894 | -2.28064 | 0.03 |
| hsa-miR-548L-002904 | 2.57148 | 1.3626 | 0.04 |
| hsa-miR-651-5p-001604 | -6.02618 | -2.59124 | 0.04 |
| mmu-miR-187-001193 | -3.54707 | -1.82663 | 0.04 |
| hsa-miR-32-002109 | 2.27452 | 1.18556 | 0.04 |
| hsa-miR-186#-002105 | -4.24328 | -2.08518 | 0.05 |
| hsa-miR-377-000566 | -4.55056 | -2.18605 | 0.05 |
| hsa-miR-139-3p-00231 | -2.35355 | -1.23484 | 0.05 |
| hsa-miR-432#-001027 | -2.16548 | -1.11468 | 0.05 |

^1^miRNA with a p-value<0.005 were carried into the internal replication phase. miRNA with a p-value <0.05 that also had a p-value of <0.05 in Supplemental Table 1 were also carried into the internal replication phase.
