# Supplementary material for: Plasma microRNA expression in adolescents and young adults with endometriosis: the importance of hormone use
Source: Front Reprod Health. 2024 Apr 11;6:1360417. doi: 10.3389/frph.2024.1360417 (PMC11043576; doi:10.3389/frph.2024.1360417)
Supplement: Supplementary file 3 [file Table3.docx]

**Supplemental Table 3**. miRNAs examined in the internal replication phase.

| **miRNA Name** | **Sequence** | **Literature References** | **Source** |
| --- | --- | --- | --- |
| hsa-miR-135a-5p | UAUGGCUUUUUAUUCCUAUGUGA | Cho FS 2015^1^ | Literature review |
| hsa-miR-451a | AAACCGUUACCAUUACUGAGUU | Cosar FS 2016;^2^ Nothnick Reprod Sci 2017;^3^ Joshi Human Reprod 2015;^4^ Graham Human Reprod 2015^5^ | Literature review |
| hsa-miR-125b-5p | UCCCUGAGACCCUAACUUGUGA | Cosar FS 2016^2^ | Literature review |
| hsa-miR-3613-5p | UGUUGUACUUUUUUUUUUGUUC | Cosar FS 2016^2^ | Literature review |
| hsa-miR-941 | CACCCGGCUGUGUGCACAUGUGC | Elias eLife 2017^6^ | Literature review |
| hsa-miR-532-5p | CAUGCCUUGAGUGUAGGACCGU | Elias eLife 2017^6^ | Literature review |
| hsa-miR-1908-5p | CGGCGGGGACGGCGAUUGGUC | Elias eLife 2017^6^ | Literature review |
| hsa-miR-30d-5p | UGUAAACAUCCCCGACUGGAAG | Elias eLife 2017^6^ | Literature review |
| hsa-miR-519d-3p | CAAAGUGCCUCCCUUUAGAGUG |  | Discovery set (non-hormone users) |
| hsa-miR-588 | UUGGCCACAAUGGGUUAGAAC |  | Discovery set (non-hormone users) |
| hsa-miR-106a-3p | CUGCAAUGUAAGCACUUCUUAC |  | Discovery set (non-hormone users) |
| hsa-let-7a-3p | CUAUACAAUCUACUGUCUUUC | Seifer Reprod Sci 2017^7^ | Discovery set (hormone users) |
| hsa-miR-127-5p | CUGAAGCUCAGAGGGCUCUGAU |  | Discovery set (non-hormone users) |
| hsa-miR-192-3p | CUGCCAAUUCCAUAGGUCACAG |  | Discovery set (non-hormone users) |
| hsa-miR-641 | AAAGACAUAGGAUAGAGUCACCUC |  | Discovery set (non-hormone users) |
| hsa-miR-548a-5p | AAAAGUAAUUGCGAGUUUUACC |  | Discovery set (non-hormone users) |
| hsa-miR-296-3p | GAGGGUUGGGUGGAGGCUCUCC |  | Discovery set (non-hormone users) |
| hsa-miR-544a | AUUCUGCAUUUUUAGCAAGUUC |  | Discovery set (non-hormone users) |
| hsa-let-7c-5p | UGAGGUAGUAGGUUGUAUGGUU | Seifer Reprod Sci 2017;^7^ Elias eLife 2017^6^ | Discovery set (non-hormone users) |
| hsa-miR-122-3p | AACGCCAUUAUCACACUAAAUA | Wang JCEM 2013^8^ | Discovery set (hormone users) |
| hsa-miR-626 | AGCUGUCUGAAAAUGUCUU |  | Discovery set (hormone users) |
| hsa-miR-29b-1-5p | GCUGGUUUCAUAUGGUGGUUUAGA |  | Discovery set (non-hormone users) |
| hsa-miR-125b-1-3p | ACGGGUUAGGCUCUUGGGAGCU |  | Discovery set (hormone users) |
| hsa-miR-147a | GUGUGUGGAAAUGCUUCUGC |  | Discovery set (non-hormone users) |
| hsa-miR-567 | AGUAUGUUCUUCCAGGACAGAAC |  | Discovery set (hormone users) |
| hsa-miR-548E-3p | AAAAACUGAGACUACUUUUGCA |  | Discovery set (non-hormone users) |
| hsa-miR-193a-3p | AACUGGCCUACAAAGUCCCAGU | Hawkins Mol Endo 2011^9^ | Discovery set (hormone users) |
| hsa-miR-154-5p | UAGGUUAUCCGUGUUGCCUUCG |  | Discovery set (hormone users) |
| hsa-miR-30c-1-3p | CUGGGAGAGGGUUGUUUACUCC |  | Discovery set (hormone users) |
| hsa-miR-541-3p | UGGUGGGCACAGAAUCUGGACU |  | Discovery set (non-hormone users) |
| hsa-miR-500a-3p | AUGCACCUGGGCAAGGAUUCUG | Cosar FS 2016^2^ | Discovery set (non-hormone users) |
| hsa-miR-548K | AAAAGUACUUGCGGAUUUUGCU |  | Discovery set (non-hormone users) |
| hsa-miR-455-5p | UAUGUGCCUUUGGACUACAUCG |  | Discovery set (hormone users) |
| hsa-miR-935 | CCAGUUACCGCUUCCGCUACCGC |  | Discovery set (hormone users) |
| hsa-miR-422a | ACUGGACUUAGGGUCAGAAGGC |  | Discovery set (hormone users) |
| hsa-miR-337-3p | CUCCUAUAUGAUGCCUUUCUUC |  | Discovery set (hormone users) |
| hsa-miR-219-1-3p | AGAGUUGAGUCUGGACGUCCCG |  | Discovery set (hormone users) |
| hsa-miR-33b-5p | GUGCAUUGCUGUUGCAUUGC |  | Discovery set (non-hormone users) |
| hsa-let-7b-3p | CUAUACAACCUACUGCCUUCCC | Cho FS 2015;^1^ Seifer Reprod Sci 2017^7^ | Discovery set (non-hormone users) |
| hsa-miR-23b-5p | UGGGUUCCUGGCAUGCUGAUUU |  | Discovery set (hormone users) |
| hsa-miR-504-5p | AGACCCUGGUCUGCACUCUAUC | Hawkins Mol Endo 2011^9^ | Discovery set (non-hormone users) |
| hsa-miR-124-3p | UAAGGCACGCGGUGAAUGCC |  | Discovery set (hormone users) |
| hsa-miR-589-5p | UGAGAACCACGUCUGCUCUGAG |  | Discovery set (hormone users) |
| hsa-miR-548i | AAAAGUAAUUGCGGAUUUUGCC |  | Discovery set (non-hormone users) |
| hsa-miR-200a-5p | CAUCUUACCGGACAGUGCUGGA | Rekker FS 2015;^10^ Hawkins Mol Endo 2011^9^ | Discovery set (non-hormone users) |
| hsa-miR-769-5p | UGAGACCUCUGGGUUCUGAGCU |  | Discovery set (non-hormone users) |
| mmu-miR-153-3p | UUGCAUAGUCACAAAAGUGAUC |  | Discovery set (non-hormone users) |
| hsa-miR-376b-3p | AUCAUAGAGGAAAAUCCAUGUU |  | Discovery set (hormone users) |
| hsa-miR-651-5p | UUUAGGAUAAGCUUGACUUUUG |  | Discovery set (both with and without hormones) |
| hsa-miR-1298-5p | UUCAUUCGGCUGUCCAGAUGUA |  | Discovery set (hormone users) |
| hsa-miR-891a-5p | UGCAACGAACCUGAGCCACUGA |  | Discovery set (hormone users) |
| hsa-miR-219a-5p | UGAUUGUCCAAACGCAAUUCU |  | Discovery set (hormone users) |
| hsa-miR-542-3p | UGUGACAGAUUGAUAACUGAAA | Wang JCEM 2013^8^ | Discovery set (hormone users) |
| hsa-miR-33a-5p | GUGCAUUGUAGUUGCAUUGCA |  | Discovery set (both with and without hormones) |
| hsa-miR-548L | AAAAGUAUUUGCGGGUUUUGUC |  | Discovery set (both with and without hormones) |
| hsa-miR-1296-5p | UUAGGGCCCUGGCUCCAUCUCC |  | Discovery set (both with and without hormones) |
| hsa-let-7i-3p | CUGCGCAAGCUACUGCCUUGCU |  | Discovery set (both with and without hormones) |
| hsa-let-7g-3p | CUGUACAGGCCACUGCCUUGC |  | Fireplex endogenous control |
| hsa-let-7d-3p | CUAUACGACCUGCUGCCUUUCU |  | Fireplex endogenous control |
| hsa-miR-29b-3p | UAGCACCAUUUGAAAUCAGUGUU |  | Fireplex endogenous control |
| ath-miR167d | UGAAGCUGCCAGCAUGAUCUGG |  | Non-human species negative control |
| oan-miR-7417-5p | UUCCCCACUCUGAGCACACAGC |  | Non-human species negative control |
| cel-miR-70-3p | UAAUACGUCGUUGGUGUUUCCAU |  | Non-human species negative control |

References

1. Cho S, Mutlu L, Grechukhina O, Taylor HS. Circulating microRNAs as potential biomarkers for endometriosis. *Fertil Steril*. 2015;103(5):1252-1260.e1. doi:10.1016/j.fertnstert.2015.02.013
2. Cosar E, Mamillapalli R, Ersoy GS, Cho SY, Seifer B, Taylor HS. Serum microRNAs as diagnostic markers of endometriosis: a comprehensive array-based analysis. *Fertil Steril*. 2016;106(2):402-409. doi:10.1016/j.fertnstert.2016.04.013
3. Nothnick WB, Falcone T, Joshi N, Fazleabas AT, Graham A. Serum MIR-451a Levels Are Significantly Elevated in Women with Endometriosis and Recapitulated in Baboons (Papio anubis) with Experimentally-Induced Disease. *Reproductive Sciences*. 2017;24(8):1195-1202. doi:10.1177/1933719116681519
4. Joshi NR, Su RW, Chandramouli GVR, et al. Altered expression of microRNA-451 in eutopic endometrium of baboons (Papio anubis) with endometriosis. *Human Reproduction*. 2015;30(12):2881-2891. doi:10.1093/humrep/dev229
5. Graham A, Falcone T, Nothnick WB. The expression of microRNA-451 in human endometriotic lesions is inversely related to that ofmacrophage migration inhibitory factor (MIF) and regulates MIF expression and modulation of epithelial cell survival. *Human Reproduction*. 2015;30(3):642-652. doi:10.1093/humrep/dev005
6. Elias KM, Fendler W, Stawiski K, et al. Diagnostic potential for a serum miRNA neural network for detection of ovarian cancer. *Elife*. 2017;6:e28932. doi:10.7554/eLife.28932
7. Seifer BJ, Su D, Taylor HS. Circulating miRNAs in Murine Experimental Endometriosis: Decreased Abundance of let-7a. *Reproductive Sciences*. 2017;24(3):376-381. doi:10.1177/1933719116667228
8. Wang WT, Zhao YN, Han BW, Hong SJ, Chen YQ. Circulating microRNAs identified in a genome-wide serum microRNA expression analysis as noninvasive biomarkers for endometriosis. *Journal of Clinical Endocrinology and Metabolism*. 2013;98(1):281-289. doi:10.1210/jc.2012-2415
9. Hawkins SM, Creighton CJ, Han DY, et al. Functional microRNA involved in endometriosis. *Molecular Endocrinology*. 2011;25(5):821-832. doi:10.1210/me.2010-0371
10. Rekker K, Saare M, Roost AM, et al. Circulating miR-200-family micro-RNAs have altered plasma levels in patients with endometriosis and vary with blood collection time. *Fertil Steril*. 2015;104(4):938-946.e2. doi:10.1016/j.fertnstert.2015.06.029
