# Supplementary material for: Plasma microRNA expression in adolescents and young adults with endometriosis: the importance of hormone use
Source: Front Reprod Health. 2024 Apr 11;6:1360417. doi: 10.3389/frph.2024.1360417 (PMC11043576; doi:10.3389/frph.2024.1360417)
Supplement: Supplementary file 4 [file Table4.docx]

**Supplemental Table 4**. Multiple comparison adjusted t-tests comparing cases vs. controls in the internal replication phase training set

| **miRNA** | **Controls (n=72) Mean (SD)** | **Cases**  **(n=36) Mean (SD)** | **Fold change** | **p-value** | **Multiple comparison adjusted**  **p-value** |
| --- | --- | --- | --- | --- | --- |
| hsa-let-7d-3p (MFI) log10 | 3.07 (0.37) | 2.89 (0.41) | 0.66 | 0.02 | 0.40 |
| hsa-miR-1908-5p (MFI) log10 | 1.16 (0.45) | 0.78 (0.95) | 0.42 | 0.03 | 0.40 |
| hsa-miR-30c-1-3p (MFI) log10 | 0.71 (0.93) | 0.29 (1.16) | 0.37 | 0.04 | 0.40 |
| hsa-miR-33a-5p (MFI) log10 | 2.03 (0.54) | 1.79 (0.65) | 0.57 | 0.04 | 0.40 |
| hsa-let-7b-3p (MFI) log10 | 1.55 (0.35) | 1.38 (0.47) | 0.67 | 0.05 | 0.40 |
| hsa-miR-23b-5p (MFI) log10 | 0.76 (0.80) | 0.27 (1.45) | 0.32 | 0.06 | 0.40 |
| hsa-miR-769-5p (MFI) log10 | 1.55 (0.43) | 1.37 (0.56) | 0.66 | 0.07 | 0.40 |
| hsa-miR-548i (MFI) log10 | -0.91 (1.25) | -1.40 (1.40) | 0.32 | 0.07 | 0.40 |
| hsa-let-7i-3p (MFI) log10 | -0.12 (0.96) | -0.50 (1.20) | 0.42 | 0.08 | 0.40 |
| hsa-let-7a-3p (MFI) log10 | 0.07 (0.47) | -0.24 (0.99) | 0.49 | 0.08 | 0.40 |
| hsa-miR-542-3p (MFI) log10 | -1.67 (1.30) | -2.12 (1.27) | 0.35 | 0.09 | 0.40 |
| hsa-miR-124-3p (MFI) log10 | -2.70 (0.91) | -2.35 (1.16) | 2.26 | 0.09 | 0.40 |
| hsa-miR-504-5p (MFI) log10 | -0.83 (1.32) | -1.30 (1.37) | 0.34 | 0.09 | 0.40 |
| hsa-miR-33b-5p (MFI) log10 | 0.78 (0.84) | 0.41 (1.23) | 0.43 | 0.11 | 0.42 |
| hsa-miR-548k (MFI) log10 | -0.01 (1.17) | -0.42 (1.38) | 0.39 | 0.11 | 0.42 |
| hsa-miR-29b-3p (MFI) log10 | 2.57 (0.35) | 2.45 (0.40) | 0.76 | 0.11 | 0.42 |
| hsa-miR-154-5p (MFI) log10 | 2.17 (0.56) | 1.99 (0.56) | 0.67 | 0.13 | 0.43 |
| hsa-miR-193a-3p (MFI) log10 | -0.16 (0.96) | 0.12 (0.78) | 1.91 | 0.13 | 0.43 |
| hsa-miR-1296-5p (MFI) log10 | 0.82 (0.92) | 0.47 (1.22) | 0.45 | 0.14 | 0.43 |
| hsa-miR-589-5p (MFI) log10 | 1.53 (0.34) | 1.42 (0.44) | 0.78 | 0.16 | 0.47 |
| hsa-miR-376b-3p (MFI) log10 | 1.21 (0.84) | 0.96 (0.96) | 0.57 | 0.18 | 0.50 |
| hsa-miR-337-3p (MFI) log10 | -0.48 (1.54) | -0.88 (1.55) | 0.39 | 0.20 | 0.54 |
| hsa-miR-641 (MFI) log10 | 0.22 (1.25) | -0.08 (1.36) | 0.50 | 0.25 | 0.65 |
| hsa-miR-500a-3p (MFI) log10 | 1.03 (0.49) | 0.84 (1.02) | 0.64 | 0.29 | 0.67 |
| hsa-miR-135a-5p (MFI) log10 | -1.94 (1.26) | -2.20 (1.21) | 0.55 | 0.31 | 0.67 |
| hsa-miR-200a-5p (MFI) log10 | -1.34 (1.34) | -1.61 (1.32) | 0.54 | 0.32 | 0.67 |
| hsa-miR-127-5p (MFI) log10 | -0.04 (0.99) | -0.24 (1.12) | 0.62 | 0.33 | 0.67 |
| hsa-miR-567 (MFI) log10 | -1.53 (1.32) | -1.78 (1.20) | 0.56 | 0.33 | 0.67 |
| hsa-miR-125b-5p (MFI) log10 | -0.11 (0.62) | -0.27 (0.90) | 0.69 | 0.34 | 0.67 |
| hsa-miR-541-3p (MFI) log10 | -2.23 (1.22) | -2.46 (1.10) | 0.59 | 0.35 | 0.67 |
| hsa-miR-219-1-3p (MFI) log10 | -1.93 (1.37) | -1.67 (1.44) | 1.85 | 0.35 | 0.67 |
| hsa-miR-3613-5p (MFI) log10 | -1.53 (1.22) | -1.76 (1.33) | 0.59 | 0.37 | 0.67 |
| hsa-miR-651-5p (MFI) log10 | -1.90 (1.28) | -1.67 (1.24) | 1.69 | 0.38 | 0.67 |
| hsa-miR-192-3p (MFI) log10 | -0.47 (1.20) | -0.27 (1.20) | 1.60 | 0.40 | 0.67 |
| hsa-miR-30d-5p (MFI) log10 | 2.52 (0.41) | 2.45 (0.43) | 0.85 | 0.41 | 0.67 |
| hsa-let-7c-5p (MFI) log10 | 2.98 (0.28) | 2.92 (0.37) | 0.88 | 0.42 | 0.67 |
| hsa-miR-296-3p (MFI) log10 | -1.97 (1.32) | -1.75 (1.40) | 1.67 | 0.42 | 0.67 |
| hsa-miR-891a-5p (MFI) log10 | -1.48 (1.58) | -1.73 (1.49) | 0.56 | 0.43 | 0.67 |
| hsa-miR-451a (MFI) log10 | 3.75 (0.11) | 3.76 (0.10) | 1.04 | 0.44 | 0.67 |
| hsa-miR-122-3p (MFI) log10 | -2.04 (1.24) | -1.86 (1.32) | 1.53 | 0.48 | 0.71 |
| hsa-miR-106a-3p (MFI) log10 | -1.91 (1.26) | -2.08 (1.16) | 0.68 | 0.51 | 0.74 |
| hsa-let-7g-3p (MFI) log10 | -0.24 (1.20) | -0.09 (1.04) | 1.42 | 0.51 | 0.74 |
| hsa-miR-1298-5p (MFI) log10 | -1.97 (1.24) | -2.11 (1.22) | 0.73 | 0.59 | 0.82 |
| hsa-miR-519d-3p (MFI) log10 | -1.60 (1.35) | -1.46 (1.26) | 1.38 | 0.61 | 0.82 |
| mmu-miR-153-3p (MFI) log10 | -1.30 (1.34) | -1.44 (1.33) | 0.73 | 0.62 | 0.82 |
| hsa-miR-588 (MFI) log10 | -1.71 (1.32) | -1.58 (1.32) | 1.34 | 0.64 | 0.82 |
| hsa-miR-29b-1-5p (MFI) log10 | -0.2 (1.21) | -0.31 (1.21) | 0.77 | 0.65 | 0.82 |
| hsa-miR-455-5p (MFI) log10 | -1.37 (1.29) | -1.48 (1.24) | 0.77 | 0.67 | 0.83 |
| hsa-miR-219a-5p (MFI) log10 | -0.63 (1.33) | -0.52 (1.11) | 1.28 | 0.68 | 0.83 |
| hsa-miR-548a-5p (MFI) log10 | -1.8 (1.24) | -1.71 (1.3) | 1.24 | 0.72 | 0.85 |
| hsa-miR-935 (MFI) log10 | -1.64 (1.36) | -1.73 (1.29) | 0.80 | 0.73 | 0.85 |
| hsa-miR-532-5p (MFI) log10 | 2.22 (0.35) | 2.19 (0.34) | 0.95 | 0.75 | 0.86 |
| hsa-miR-544a (MFI) log10 | -2.43 (1) | -2.37 (1.07) | 1.15 | 0.77 | 0.86 |
| hsa-miR-626 (MFI) log10 | -1.88 (1.24) | -1.81 (1.38) | 1.19 | 0.77 | 0.86 |
| hsa-miR-125b-1-3p (MFI) log10 | -1.75 (1.29) | -1.81 (1.29) | 0.87 | 0.82 | 0.90 |
| hsa-miR-941 (MFI) log10 | 0.79 (0.48) | 0.78 (0.41) | 0.97 | 0.87 | 0.93 |
| hsa-miR-147a (MFI) log10 | -1.64 (1.32) | -1.68 (1.31) | 0.92 | 0.89 | 0.93 |
| hsa-miR-548e-3p (MFI) log10 | -0.83 (1.26) | -0.79 (1.28) | 1.08 | 0.90 | 0.93 |
| hsa-miR-422a (MFI) log10 | 1.07 (0.7) | 1.05 (0.71) | 0.96 | 0.91 | 0.93 |
| hsa-miR-548l (MFI) log10 | -1.27 (1.4) | -1.25 (1.4) | 1.06 | 0.93 | 0.93 |
