# Supplementary figures and images for: Plasma microRNA expression in adolescents and young adults with endometriosis: the importance of hormone use
Source: Front Reprod Health. 2024 Apr 11;6:1360417. doi: 10.3389/frph.2024.1360417 (PMC11043576; doi:10.3389/frph.2024.1360417)

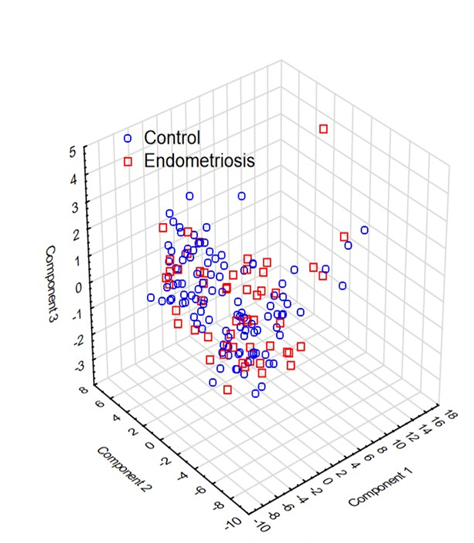

Supplement: Supplemental Figure 1 — Principal component analysis of endometriosis cases versus controls in the internal replication phase using the 63 miRNAs. [file Image1.png]

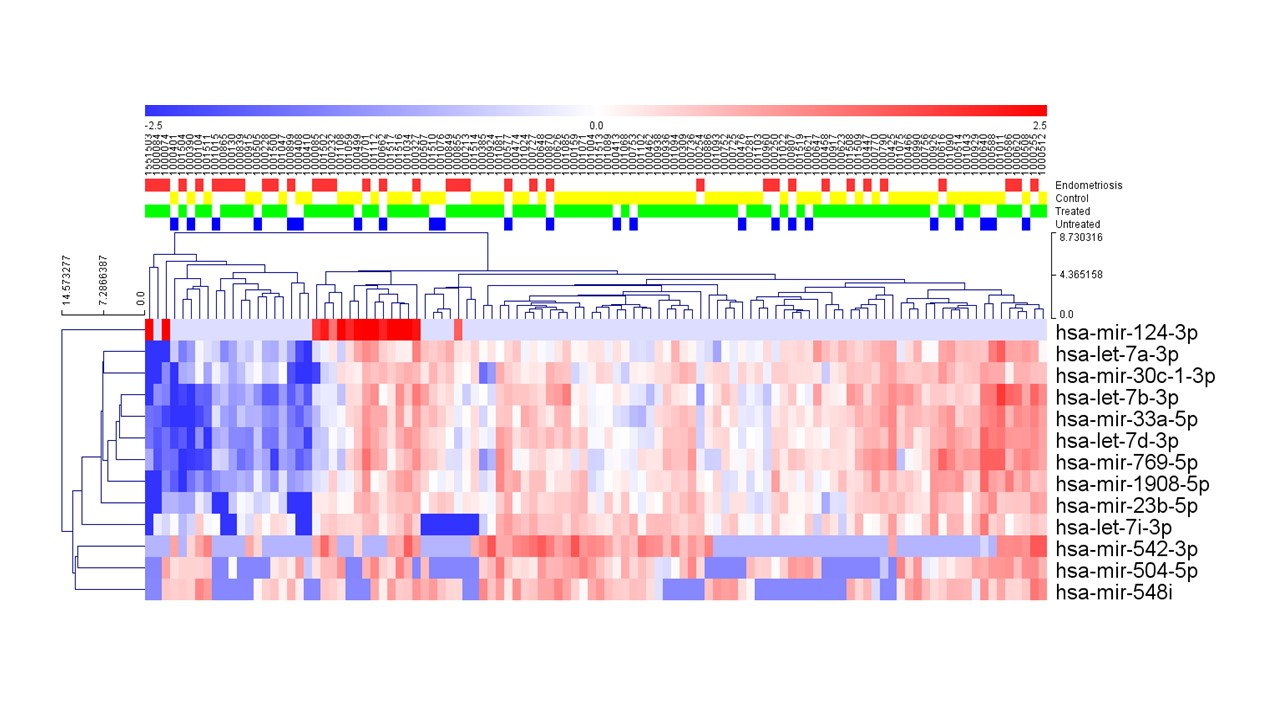

Supplement: Supplemental Figure 2 — Sample classification for miRNA in the internal replication phase. Heatmap shows sample classification for all miRNAs with a nominal p < 0.10. Data represent hierarchical clustering by Euclidean distance normalized by row and optimized for layout. [file Image2.png]
